# Characterization of host proteins interacting with the lymphocytic choriomeningitis virus L protein

**DOI:** 10.1371/journal.ppat.1006758

**Published:** 2017-12-20

**Authors:** Kseniya Khamina, Alexander Lercher, Michael Caldera, Christopher Schliehe, Bojan Vilagos, Mehmet Sahin, Lindsay Kosack, Anannya Bhattacharya, Peter Májek, Alexey Stukalov, Roberto Sacco, Leo C. James, Daniel D. Pinschewer, Keiryn L. Bennett, Jörg Menche, Andreas Bergthaler

**Affiliations:** 1 CeMM Research Center for Molecular Medicine of the Austrian Academy of Sciences, Lazarettgasse, Vienna, Austria; 2 University of Basel, Department of Biomedicine–Haus Petersplatz, Division of Experimental Virology, Basel, Switzerland; 3 Division of Protein and Nucleic Acid Chemistry, Medical Research Council Laboratory of Molecular Biology, Cambridge, United Kingdom; Division of Clinical Research, UNITED STATES

## Abstract

RNA-dependent RNA polymerases (RdRps) play a key role in the life cycle of RNA viruses and impact their immunobiology. The arenavirus lymphocytic choriomeningitis virus (LCMV) strain Clone 13 provides a benchmark model for studying chronic infection. A major genetic determinant for its ability to persist maps to a single amino acid exchange in the viral L protein, which exhibits RdRp activity, yet its functional consequences remain elusive. To unravel the L protein interactions with the host proteome, we engineered infectious L protein-tagged LCMV virions by reverse genetics. A subsequent mass-spectrometric analysis of L protein pulldowns from infected human cells revealed a comprehensive network of interacting host proteins. The obtained LCMV L protein interactome was bioinformatically integrated with known host protein interactors of RdRps from other RNA viruses, emphasizing interconnected modules of human proteins. Functional characterization of selected interactors highlighted proviral (DDX3X) as well as antiviral (NKRF, TRIM21) host factors. To corroborate these findings, we infected *Trim21*^-/-^ mice with LCMV and found impaired virus control in chronic infection. These results provide insights into the complex interactions of the arenavirus LCMV and other viral RdRps with the host proteome and contribute to a better molecular understanding of how chronic viruses interact with their host.

## Author summary

RNA-dependent RNA-polymerases (RdRps) play a key role in the life cycle of RNA viruses. They interact with cellular proteins during replication and transcription processes and impact the immunobiology of viral infections. This study characterized the host protein interactome of the RdRp-containing L protein of the prototypic arenavirus lymphocytic choriomeningitis virus (LCMV). Several L protein interactors with proviral and antiviral effects were identified *in vitro*, and mice lacking the identified L protein interactor TRIM21 exhibited impaired control of chronic LCMV infection. Integration of the L protein interactomes with known RdRp interactomes from other RNA viruses highlighted common and virus-specific strategies to interact with the host proteome, which may indicate novel avenues for antiviral interventions.

## Introduction

RNA viruses hijack and utilize host factors at all steps of their life cycle. The viral RNA-dependent RNA polymerase (RdRp) is a key enzyme responsible for transcription and replication of viral genomes. Three-dimensional complete protein structures are available for RdRps from different RNA viruses and feature a similar “right handed” structure containing six conserved motifs [1–3]. The high similarity of RdRps between different viruses suggests that they might interact with similar host factors in the viral life cycle. Global host–virus protein interaction networks provided invaluable insights into infection biology [4–8], and similar approaches hold promise for an in-depth understanding of how viral RdRps interact with the host.

Lymphocytic choriomeningitis virus (LCMV) is a (-) RNA virus of the *Arenaviridae* and represents a well-established model system that led to seminal findings in immunology and host-pathogen research [9–12]. Particularly, it serves as a benchmark model of chronic viral infections [9, 13] whereby the strain Clone 13 (Cl13) causes immunosuppression and persists in mice for several months [14, 15]. The genetically closely related LCMV strain Armstrong (ARM) leads to acute infection, which is efficiently cleared from the blood of infected mice within a week [14, 15]. A single amino acid exchange at the position 1079 of the L protein (K1079Q) was found to be required for the ability of Cl13 to establish persistence with additional contribution of the mutation F260L in the viral glycoprotein [14–16]. The L gene encodes the largest open reading frame (250 kDa) in the LCMV genome, which together with the viral nucleoprotein (NP) forms the ribonucleoprotein complex (RNP) required for viral transcription and replication [17, 18]. The L protein contains several domains of which the NL1 domain serves as a type II endonuclease and domain III possesses RdRp activity [19, 20]. Yet, a better molecular understanding of the L protein and its role in the viral biology is hampered by lack of complete protein structure and unknown host protein interactors.

In this study we set out to determine the host interactome of the LCMV Cl13 L protein to gain insights into the interactions of the LCMV replication complex with host factors and to investigate the molecular environment that modulates viral immunobiology. We developed infectious LCMV virions with a protein tag fused to the L protein, enabling us to identify interacting host proteins by immunoprecipitation followed by mass spectrometry. Functional characterization of selected interaction partners *in vitro* revealed the impact of several interactors on LCMV replication. This was corroborated in mice lacking one of these interactors–TRIM21, which exhibited impaired control of chronic LCMV infection. Finally, we integrated previously reported interactomes from other viruses by a global network analysis and identified common and unique highly interconnected cellular protein functional modules targeted by different viral RdRps.

## Results

### Generation and characterization of LCMV strains expressing a tagged L protein

**Fig 1:**
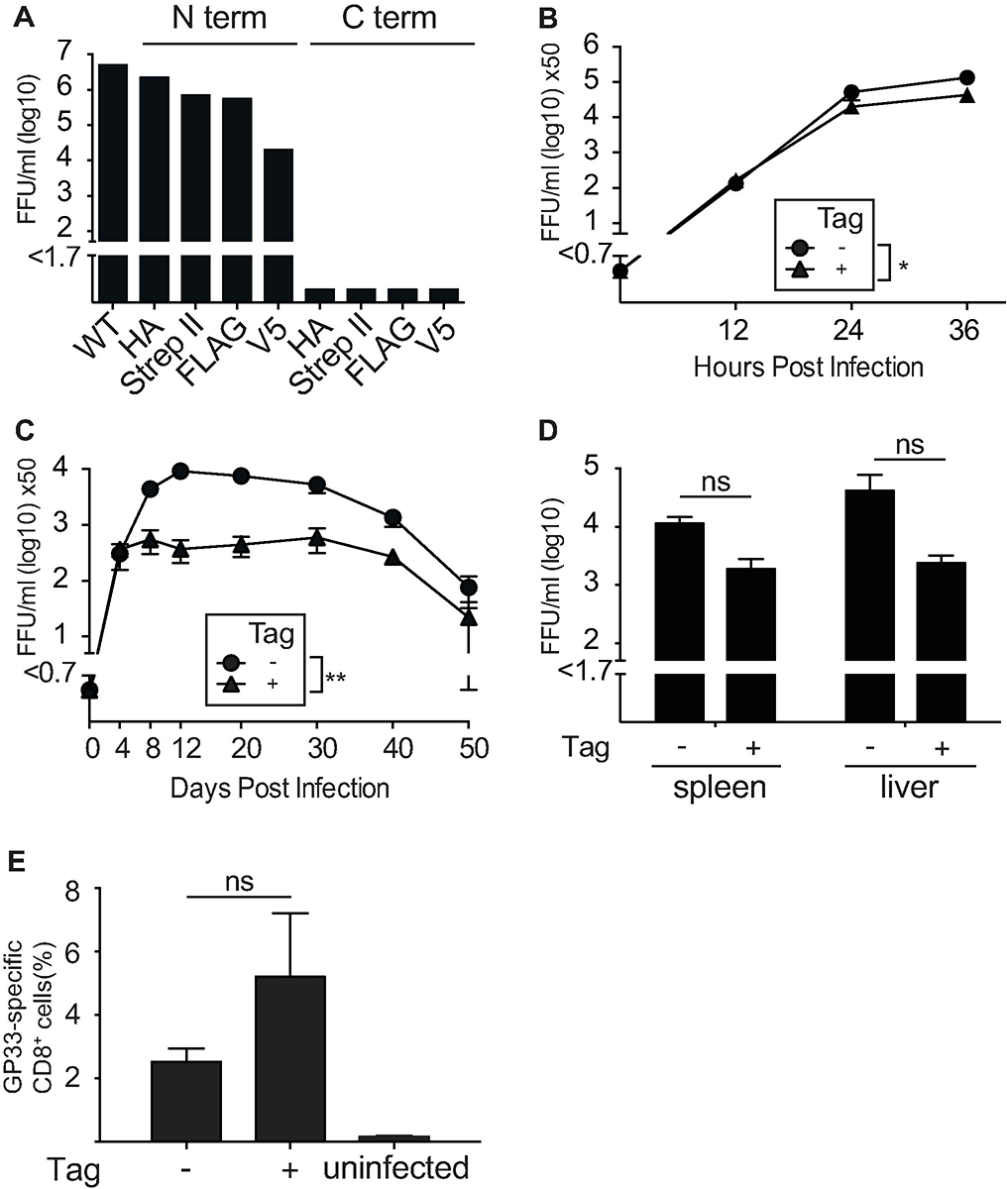
Generation and characterization of LCMV strains expressing a tagged L protein. **(A)** Viral titer of N- and C-terminal L protein-tagged Cl13 LCMV and WT Cl13 LCMV measured by focus forming assay at 72 hours post infection after reverse genetic rescue on BHK21 cells. (**B**) HEK293T cells were infected at a MOI of 0.01 with either Cl13_L-HA_ or with untagged Cl13. Supernatant was harvested and viral loads were measured at the indicated time points by focus forming assay. **(C** and **D)** C57BL/6J mice were infected with 2x10^6^ FFU of the indicated viruses. Viral titers were determined in **(C)** blood at indicated time points and in **(D)** organs 20 days post infection. **(E)** C57BL/6J mice were infected with 2x10^6^ FFU of the indicated viruses and the percentage of GP33-specific-tetramer^+^ CD8^+^ T cells was quantified in the spleen at 8 days post infection. Each symbol and bar represents the mean ± SEM of three to five mice. Statistical significance was calculated by Two-way ANOVA (B-**C**) or unpaired t-test (**D-E**). Significant p values were indicated as follows: ns—non significant, * p≤0.05,: ** p≤0.01.

To elucidate the host protein interactome of the L protein of LCMV, we developed a strategy of endogenous protein tagging that enabled us to analyze the host cell interaction partners in the context of the natural infectious life cycle. Arenaviruses have a compact genome and even minor modifications can severely affect their viability. We, thus, selected four different affinity purification tags with a short size of 8 to 14 amino acid residues (HA, Strep II, FLAG and V5) and introduced the tags either on the N- or C- terminus of the L ORF by insertional cDNA mutagenesis (**S1 Table**). Reverse genetic rescue of the corresponding viruses revealed that all viruses bearing an N terminally tagged L protein were efficiently generated on a Cl13 LCMV backbone while none of the C terminally tagged L protein constructs resulted in infectious virions (**Fig 1A**). This demonstrated that it was feasible to generate L protein tagged LCMV variants with retained infectivity in cell culture. Based on the viral titers recovered, we continued to work with a LCMV Cl13 virus whose L protein was N terminally fused with a HA tag (Cl13_L-HA_). To control for the expression of the tagged L protein, we prepared lysates from cells infected with Cl13_L-HA_ and detected the L protein at the expected size by anti-HA antibody (**S1A Fig**). To assess the effect of the introduced tag on viral propagation, we compared the growth kinetics of Cl13_L-HA_ to an untagged Cl13 virus upon infection of HEK293T cells. This experiment revealed exponential growth of Cl13_L-HA_ albeit with slight attenuation compared to the untagged Cl13 virus (**Fig 1B**).

To assess the fitness of the LCMV variant with tagged L protein *in vivo*, we infected mice with Cl13_L-HA_ or the corresponding untagged Cl13 virus and assessed viral loads in blood (**Fig 1C**), spleen and liver (**Fig 1D**). The virus encoding the HA-tagged L protein showed a similar kinetics of viremia compared to the untagged virus, although peak titers were lower when mice were infected with Cl13_L-HA_ virus. Quantification of virus-specific CD8^+^ T cell responses showed no significant differences between mice infected with either Cl13_L-HA_ or untagged Cl13 (**Fig 1E**). To assess the stability of the HA tag in the LCMV genome, we sequenced virus from the spleen lysates of mice which had been infected with either Cl13_L-HA_ or untagged Cl13 50 days previously. These results confirmed the presence of the HA tag and did not provide any evidence for acquired mutations in the corresponding region at this late time point (**S1B Fig**).

Together, these data indicated that Cl13_L-HA_ has similar immunobiological characteristics as the respective untagged Cl13 strain. These novel tools enabled us to proceed with immunoprecipitation of the L protein in the context of a natural viral infection.

### Identification of the LCMV L protein interactome

**Fig 2:**
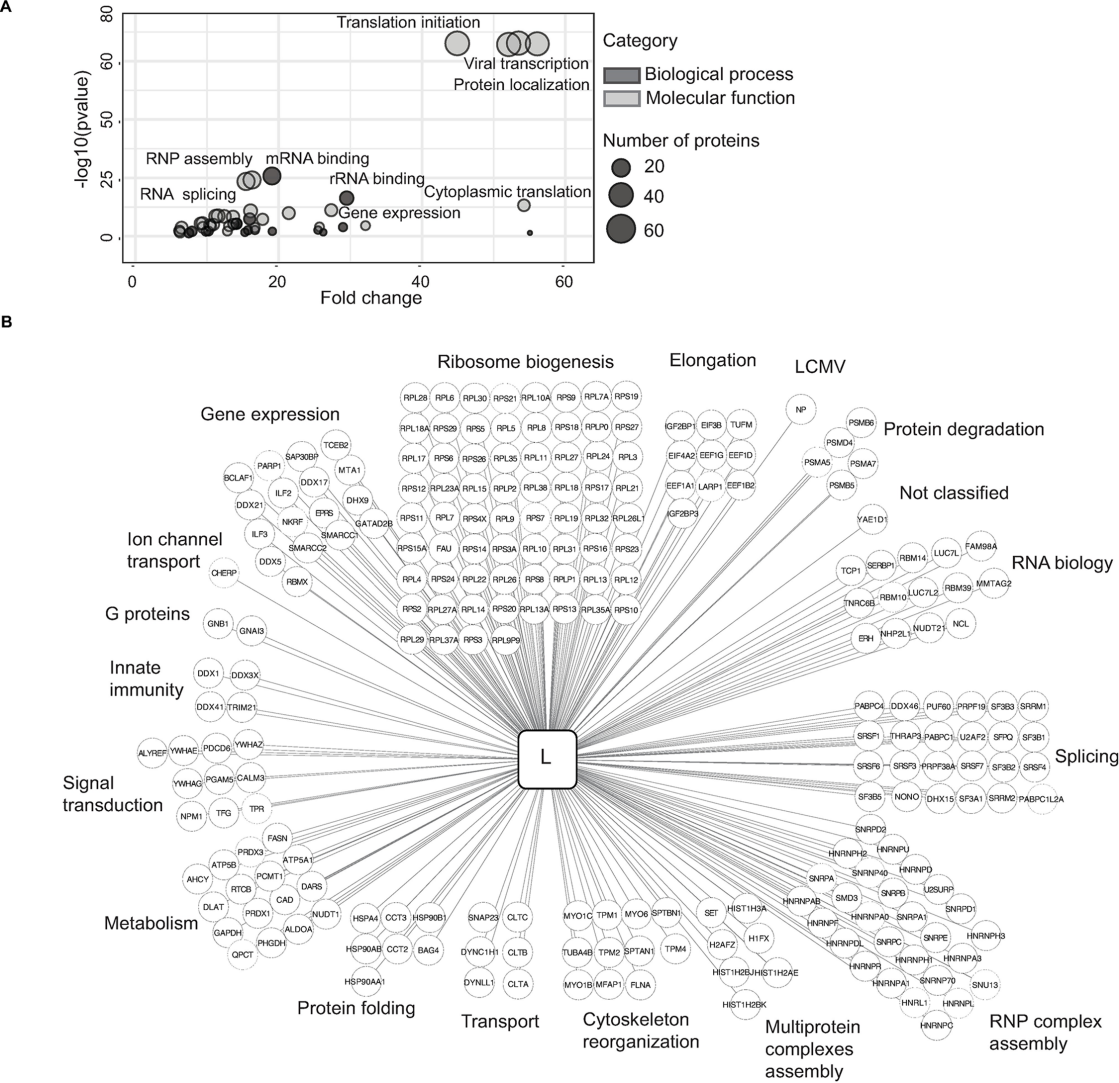
Identification of L protein interactome. **(A)** GO enrichment analyses for the L protein interactome based on the molecular functions (light grey) and biological processes (dark grey) of interactors followed by visualization with ReviGO. **(B)** Overview of L protein interactomes classified based on the protein functions and visualized in Cytoscape. The data is based on the mass-spectrometry derived list of proteins identified in L protein pulldowns after filtration using Top3 quantitation and SAINTexpress software as detailed in Materials and Methods.

For the identification of the LCMV L protein interactome, we relied on a well-established cell culture system [21]. HEK293T FlpIn cells infected with Cl13_L-HA_ were used for mass spectrometry analyses. In addition, we infected HA-GFP overexpressing as well as wild type HEK293T FlpIn cells with untagged Cl13 virus which served as negative controls for the interactome analysis. Subsequently, we performed an one-step anti-HA immunoprecipitation from infected cell lysates (**S1C Fig**), and analyzed interaction partners of the LCMV L protein by one-dimensional gel-free liquid chromatography tandem mass spectrometry (LC–MS/MS). In total we identified 555 proteins in the L protein pulldowns. Upon stringent filtration against the background of our control samples (**Materials and Methods**), we obtained 231 interactors for our bait (**Fig 2, S2 Table**). Our bioinformatic analysis, which was based on global gene set enrichment and functional classification, revealed that the major components of the LCMV L protein interactome are RNA biology-associated proteins (e.g. splicing, ribonucleoproteins, ribosomal and RNA-binding proteins) and proteins related to translation and transcription (**Fig 2A and 2B**). Importantly, the LCMV nucleoprotein (NP), a known interactor of the L protein [17], was detected in all L protein pulldowns (2 biological replicates and 2 technical replicates each) but not in our control samples infected with untagged Cl13 virus, serving as a valuable internal control (**Fig 2B, S1C Fig**). In addition to the identified global clusters depicted in **Fig 2A**, we found a number of interactors with reported roles in signal transduction and innate immunity (**Fig 2B**). These included DEAD-box helicase 3 (DDX3X), insulin-like growth factor 2 (IGF2BP1), NF-kB repressing factor (NKRF) and Tripartite Motif Containing 21 (TRIM21). To confirm selected host protein interactions for the L protein, we used available antibodies specific to the endogenous interaction partners DDX3X, polyadenylate-binding protein 1 (PABPC1) and TRIM21 and performed western blots on anti-HA immunoprecipitates of Cl13_L-HA_-infected HEK293T cell lysates (**S1D Fig**). The L protein of the acute LCMV strain ARM differs in the single amino acid exchange K1079L and plays a key role in viral immunobiology [14–16]. We, thus, generated also an LCMV variant expressing the HA-tagged L protein of ARM to perform co-immunoprecipitation experiments. These revealed that pulldown of the ARM L protein enriched for DDX3X, PABPC1 and TRIM21 as well, indicating that the interactions are not affected by the mutation K1079L (**S1D Fig**). To provide additional evidence for direct binding of these host proteins to the L protein in the absence of viral RNA or other viral proteins, we transiently transfected cells with a plasmid encoding the HA-tagged L protein of Cl13. In line with our previous findings, HA-specific immunoprecipitation confirmed DDX3X, PABPC1 and TRIM21 as L protein interactors (**S1E Fig**). We also performed a reciprocal co-immunoprecipitation experiment by transfecting HEK293T cells with plasmids encoding HA-tagged L protein and/or V5-tagged TRIM21 (**S1F Fig**), corroborating the interaction between the viral L protein and TRIM21.

Together, our experiments with endogenously-tagged infectious LCMV characterized the host protein interactome of the LCMV L protein.

### Viral RNA-dependent RNA-polymerases target the host proteome by common and virus-specific strategies

**Fig 3:**
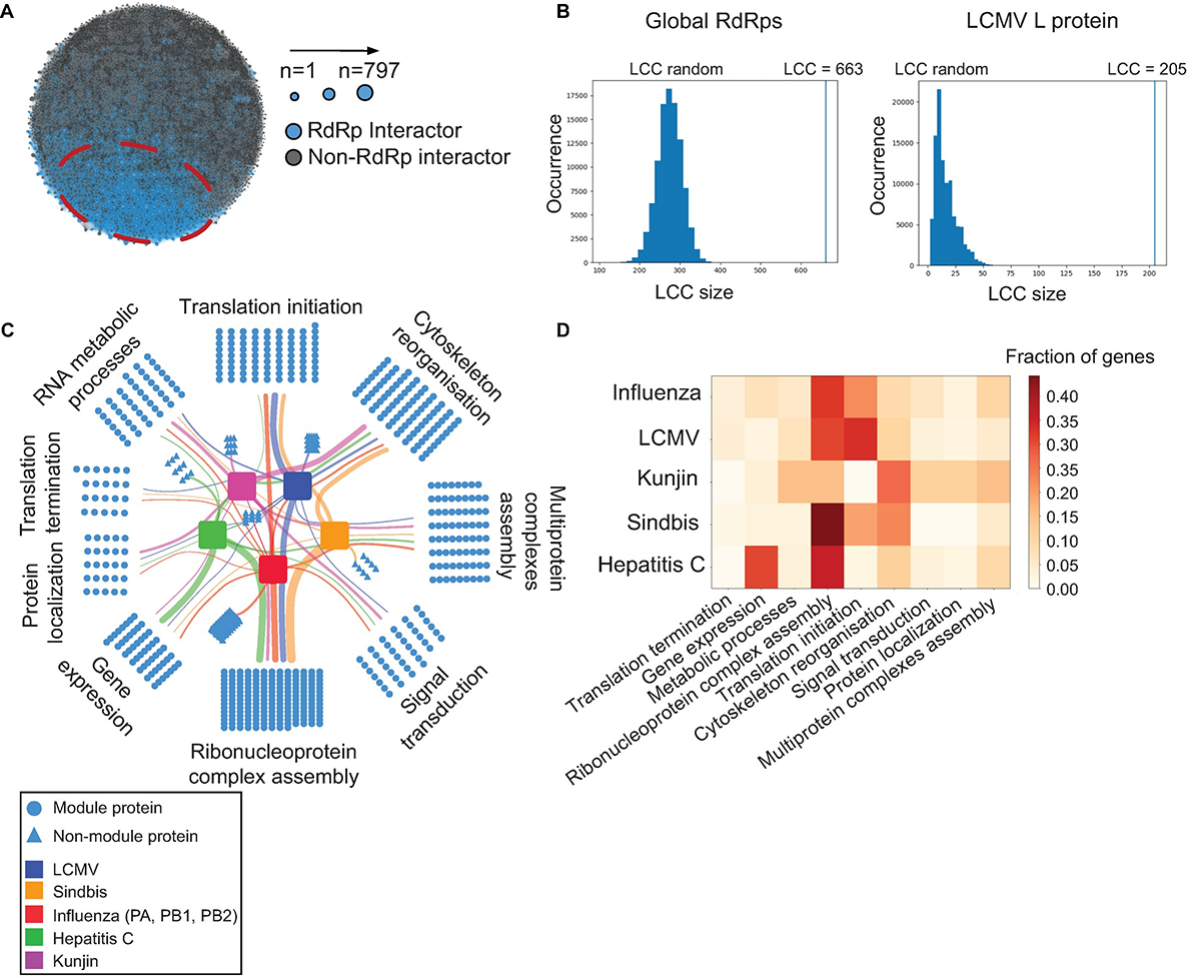
Viral RNA-dependent RNA-polymerases target host proteome by common and virus-specific strategies. **(A)** Integrated interactome of viral RdRp targets. Host proteins interacting with viral RdRps are highlighted in blue, the rest of the human proteome—in grey. **(B)** Largest connected component (LCC) analyses for global RdRps and LCMV only datasets. **(C)** Functional protein modules targeted by RdRps based on the community detection method. **(D)** Heat map representing virus-specific targeting of protein functional modules.

To better understand the global landscape of interactions between viral replication machineries and the host, we integrated the LCMV L protein interactome obtained in this study with publicly available interactome datasets on other RNA viruses (**S3 Table**) [22], resulting in a comprehensive network of all hitherto known interactomes of viral RdRps (**Fig 3A**). We then mapped this host-RdRp network onto the global human interactome consisting of 141,296 experimentally verified physical interactions between 13,460 proteins [23], thus allowing us to locate all human proteins targeted by various RdRps. Proteins associated with the same disease have been found to aggregate in the same local neighborhood of the human interactome [23]. To test whether proteins targeted by RNA viruses display a similar tendency we analyzed the size of the largest connected component (LCC) [24], i.e. the number of directly connected proteins (see Methods and **Fig 3A**). Out of 797 host proteins targeted by various RdRps, 663 proteins are directly connected to each other by protein-protein interactions, suggesting that indeed viruses affect a specific neighborhood of the human interactome. The obtained LCC values were significantly higher than the random expectation both for global RdRps (p-value = 1.47e-36) and for LCMV Cl13 L protein only (p value = 9.77e-84) datasets (**Fig 3B**), indicating that viral RdRps specifically target highly interconnected host protein interactome modules.

To further elucidate the biological function of the targeted interactome neighborhood we performed a gene ontology (GO) enrichment analyses and clustered the targets based on their functional similarity using a network-based community detection method [25]. We identified 9 basic functional protein modules in the human proteome that are targeted by various viral RdRps (**Fig 3C–3D**). While most of them are associated with RNA biology-related processes such as RNA biosynthesis, RNA metabolic processes and ribonucleoprotein complex assembly, we also identified other cellular processes including cytoskeleton reorganization and signal transduction. Interestingly, certain protein modules are commonly targeted by all viruses (e.g. ribonucleoprotein complex assembly) whereas other modules seem to be targeted in a virus-specific manner (**Fig 3D**). These analyses pinpointed that viral RdRps target the host proteome by common and virus-specific strategies.

### Functional screening for L protein interactors involved in LCMV infection

In order to elucidate a potential functional role of the identified host factors in LCMV infection, we selected the five proteins DDX3X, FLII, IGF2BP1, NKRF and TRIM21 from the total list of interactors. TRIM21 and DDX3X were among the proteins that are targeted by the polymerase complexes of several viruses [8, 21], which suggested that these host factors may play an important role in the replication of RNA viruses. The other host proteins were selected based on their known immune functions or their role in the life cycle of other viruses. We designed CRISPR-Cas9 assays for the selected candidates and created two independent cell pools by individual single guide RNA per gene. These targeted cell pools and non-target control cells were generated in HeLa S3 cells and pools of early passage 1 or 2 were used. This human cell line supports LCMV replication and mounts an antiviral innate immune response. Specific gene editing was confirmed by T7EI cleavage assay (**S2A Fig)** and TIDE approach **(S2B Fig)** for the targeted cells. Further, we confirmed the decreased protein expression of DDX3X and TRIM21 in the respective targeted cells by western blot (**S2C Fig**). We also attempted to generate cells targeting the validated L interactor PABPC1, but were unsuccessful, possibly because PABPC1 is an essential gene *[26]*.

**Fig 4:**
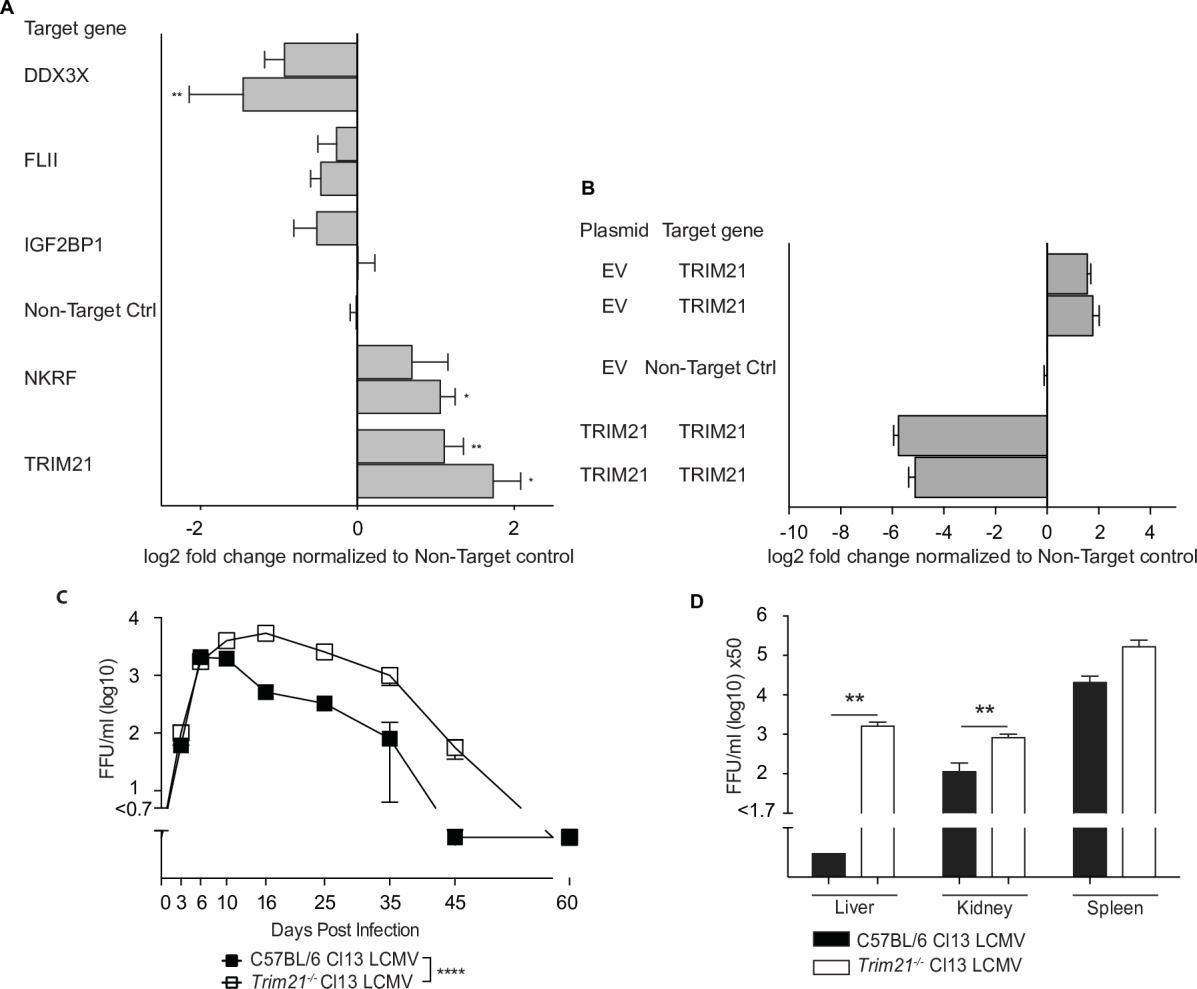
Functional screening for L protein interactors involved in LCMV life cycle. (**A**) Two independently generated HeLa S3 CRISPR-Cas9 targeted cell pools per gene of interest for 5 genes were infected in triplicate wells with LCMV Cl13 WT at a MOI of 0.01 and viral loads were measured at 36 hours post infection by focus forming assay. The obtained data were normalized to the non-target control and log2 transformed. (**B**) Two HeLa S3 CRISPR-Cas9 TRIM21-targeted cell pools were reconstituted either with TRIM21-expressing plasmid or with non-target control and 36 hour post transfection were infected in triplicate wells with LCMV Cl13 WT at a MOI of 0.01. Viral loads were measured at 36 hours post infection by focus forming assay. The obtained data were normalized to the non-target control and log2 transformed. (**C**-**D**) C57BL/6 and *Trim21*^-/-^ mice were infected with 2x10^6^ FFU of the indicated viruses. Viral titers were determined in (**C**) blood at indicated time points and in (**D**) organs at 21 days post infection. The data shown in (**C**) is representative of two similar experiments. Each symbol and bar represents the mean ± SEM of three to five mice. Statistical significance was calculated by unpaired t-test (**A, B, D**) or by Two-way ANOVA (**C**). Significant p values were indicated as follows: ns—non significant, * p≤0.05, ** p≤0.01, *** p≤0.001, **** p≤0.0001.

The obtained targeted cells were subsequently infected with Cl13 WT and viral propagation was measured by focus forming assay (FFA) (**Fig 4A**). These experiments revealed that Cl13 replicated to higher titers in TRIM21-targeted and NKRF-targeted cells. Conversely, we found reduced viral loads in DDX3X-targeted cells, which is in line with its proviral effect in HCV infection [27]. Other targeted cells did not reveal consistent differences in viral loads, which could reflect functional redundancies or cell type-specific properties. We also determined the effect of LCMV infection on the expression levels of TRIM21 in infected HEK293T cells and found, that it is not significantly changed upon infection (**S3 Fig**).

To further validate the findings of our functional screen, we performed reconstitution experiments with TRIM21. TRIM21-targeted cells were transfected with either a plasmid expressing TRIM21 or with an empty vector (EV) control plasmid and subsequently infected with Cl13 WT. Importantly, reconstitution with TRIM21 reversed the phenotype previously observed in TRIM21-targeted cells and led to reduced viral titers (**Fig 4B**). Similar antiviral effects of TRIM21 were observed upon infection with ARM WT (**S4A Fig**).

Finally, we infected *Trim21*^-/-^ and WT mice with Cl13 WT to assess viral propagation *in vivo* [28]. Interestingly, *Trim21*^-/-^ and WT mice showed comparable levels of viremia in the early stage of infection but approximately two weeks after infection *Trim21*^-/-^ mice exhibited impaired control of Cl13 in the blood compared to WT mice (**Fig 4C,** p-value <0.0001). Similarly, we found higher viral loads in spleen, liver and kidney of infected *Trim21*^-/-^ mice compared to WT mice (**Fig 4D**). We also infected *Trim21*^-/-^ and WT mice with the acute strain ARM but were unable to detect differences in viral loads in blood and other organs (**S4B and S4C Fig**). This was in line with comparable viremia kinetics of Cl13 in *Trim21*^-/-^ and WT mice during the early phase of infection.

In summary, these experiments provided novel insights into the impact of identified L protein interactors on the life cycle of LCMV and uncovered a non-redundant antiviral role of TRIM21 in the control of chronic LCMV infection.

## Discussion

This study gives novel insights into how the replication machinery of LCMV interacts with the host on a protein-protein level by the use of reverse genetically engineered endogenously tagged viruses. Similar to recent approaches for endogenous tagging of viral proteins [29–31], this avoids potential artifacts generated by conventional ectopic overexpression of tagged viral proteins due to non-physiological protein abundance and/or localization and may be applicable to the study of other viral protein interactomes. Moreover, it enables the proteomic characterization of viral RdRps and other viral proteins with cellular partners not only in cell lines but also in more complex systems such as primary cells and tissues *ex vivo*. We acknowledge that affinity purification mass-spectrometry approaches may also yield interactors which are not directly binding to the protein-of-interest but rather to complex partners. In the case of the L protein this may include interactions with other components of the arenaviral ribonucleoprotein complex or the viral Z protein [32, 33].

Arenaviruses have compact genomes and any minor modification may severely affect infectivity. Interestingly, N-terminally L-tagged LCMV constructs resulted in infectious virions, whereas tags fused to the C terminus of the L protein prevented viral reverse genetic rescue (**Fig 1A).** This may reflect impaired protein structure-function of the L protein or possibly indicate reduced fitness due to noncoding structural effects on the viral RNA genome [34].

Our proteomic analyses demonstrate that the LCMV L protein targets multiple cellular pathways such as RNA biology-associated modules (e.g. RNA metabolic processes and ribonucleoprotein complex assembly), cytoskeleton reorganization, protein localization and translation. Interestingly, the L protein also binds to numerous host proteins associated with signal transduction and innate immune signaling. This is of interest due to the known intricate and multi-faceted relation of LCMV with the antiviral type I interferon system [35, 36].

By combining our proteomic results with loss-of-function experiments we implicated several interactors in the course of LCMV infection. As examples, the E3 ligase TRIM21 and the RNA helicase DDX3X were identified as interactors of the LCMV L protein and loss of either protein had an effect on viral propagation. TRIM21 is known to regulate the type I interferon response via ubiquitination of multiple interferon regulatory factors (IRFs) and acts as an intracellular Fc receptor [37, 38], whereas DDX3X is involved in innate immune signaling cascades [39, 40]. Of note, both TRIM21 and DDX3X are targeted by the polymerase complexes of influenza virus and HCV [8, 21]. These two proteins provide an example of host factors located at the interface of several viral RdRp interactomes. Bioinformatic analyses of integrated host-viral interactomes can highlight such “hot spots” in the human proteome for further investigations. Moreover, DDX3X was recently also described as interactor of LCMV NP [41], indicating that individual host factors may bind to several viral proteins.

Based on our mass spectrometric analysis and the loss-of-function experiments *in vitro*, we infected *Trim21*^*-/-*^ mice with LCMV strain Cl13 and found impaired virus control in the late phase of infection (**Fig 3C and 3D**). These results revealed a novel role for TRIM21 in the replication of LCMV both *in vitro* and *in vivo*. Of note, LCMV is an enveloped arenavirus whereas the antibody-mediated antiviral effects of TRIM21 have been predominantly linked to non-enveloped viruses such as adenoviruses [42], caliciviruses [38] and picornaviruses [43]. Further investigations will be required to dissect the TRIM21-dependent mechanisms for the effective control of chronic LCMV infection.

Our proteomic results provide a data-rich resource for the study of arenaviruses in general. Further, the LCMV L protein interactome may bring novel impetus to unravel the functional implications of the viral K1079Q mutation and the associated immunobiological differences between different strains of LCMV and their abilities to persist in the mouse. Finally, our integration of the LCMV L protein interactome with publicly available data of other RdRps emphasizes the impact of mass spectrometry-based proteomics on virological research [44], and provides a global overview of the host factors targeted by viral RNA polymerases. Of note, these available datasets were derived from overexpressed viral RdRps while the interactome data of this study was obtained in the context of endogenously tagged replicating viruses and may thus also contain proteins binding to the polymerase complex. Yet, the high interconnectivity within the cluster of viral targets suggests that RdRps target functionally important protein modules. This could be associated with the conserved structure and similar functional host-dependent requirement of viral RdRps. Further, our network analyses emphasize the fact that viral RdRps from different taxonomy groups target functional host protein modules in common and virus-specific ways. This may highlight RdRp-host protein interactions that are required for the majority of RNA viruses as well as reveal unique features of distinct virus species. This expanded view on the interaction of viral replication machineries with their host proteomes may contribute to the development of novel antiviral therapeutic avenues.

## Materials and methods

### Animal experiments

C57BL/6J mice were originally obtained from The Jackson Laboratory. *Trim21*^-/-^ mice were generated by gene disruption via insertion of EGFP into exons 3–5, as described [28]. Mice were subsequently rederived by transferring embryos from the original knockout C57BL/6 strain into pathogen-free recipient C57BL/6 females. Mouse experiments were performed with sex- and age-matched animals under specific pathogen-free conditions.

### Cells

All cell lines were maintained in Dulbecco׳s Modified Eagle Medium (Gibco) supplemented with 10% FCS (Invitrogen) and Penicillin-Streptomycin-Glutamine (Thermo Fisher Scientific, 10378016). HEK293 Flp-In TREx cells (Invitrogen) with doxycycline-dependent transgene expression were used to generate the cell line for Strep-HA-GFP overexpression according to the manufacture protocol. This cell line was treated for 48 h with doxycycline (1μg/mL) to induce overexpression of the tagged protein.

### Viruses and infections

L protein-tagged LCMV was generated using a reverse genetic system approach [45]. A detailed description for the generation of the N-terminal HA-tagged L protein Cl13 virus (Cl13_L-HA_) is provided below. The sequence encoding the HA tag (TATCCGTATGATGTGCCGGATTATGCG) followed by the sequence encoding a short linker GGS (GGTGGTTCT) was used to design primers for an insertional mutagenesis approach. Additionally, a single nucleotide exchange (non-coding mutation) in the second codon of L ORF (A to G) was introduced to distinguish viruses that may have lost the HA tag from wild type viruses. All primer sequences for this reverse genetic approach are described in **S1 Table**. pl-L-Cl13(-) plasmids were employed for insertional mutagenesis by PCR with Phusion High-Fidelity DNA Polymerase (Thermo Scientific, F530S). The obtained plasmid was further used to generate HA-tagged L1079K virus using insertional mutagenesis. These plasmids encoding the L-tagged segment were used to rescue corresponding viruses according to the standard reverse genetics protocol [45]. LCMV titers were determined by NP-specific focus forming assay (FFA) as described previously using Vero cells (ATCC-CCL-81) [46].

Reverse genetically rescued WT (untagged) viruses were used throughout all experiments with the exception of Fig 4C and 4D and S4B and S4C Fig. For the latter experiments with WT and *Trim21*^-/-^ mice we used passaged WT ARM and Cl13 virus stocks whose origins are described in [15].

For mass spectrometry (MS) analyses HEK293 FlpIn TREx cells (Invitrogen, R78007) were infected with Cl13_L-HA,_ negative control cells Strep-HA-GFP expressing HEK293 Flp-In TREx and empty HEK293 FlpIn TREx cells were infected with untagged Cl13 at a multiplicity of infection (MOI) of 0.01, washed and harvested with ice-cold phosphate-buffered saline (PBS) after 36 hours post infection.

Mice were infected intravenously with 2x10^6^ focus forming units (FFU) of the corresponding virus stock. To confirm the presence of the HA tag at 50 days post infection, total cellular RNA was extracted from the spleen of mice infected with either Cl13_L-HA_ or untagged Cl13 using QIAzol lysis reagent (QIAGEN, 79306) according to the standard protocol to confirm the presence of the genetic tag. cDNA was obtained with random hexamers by Superscript II RT PCR and used as an input for amplification with LCMV-specific primers (5`gtgctgtgaaagcttaccagcctatc`3 and 5`tatccgtatgatgtgccggattatg`3) by Phusion High-Fidelity DNA Polymerase (Thermo Fisher Scientific, F530S). This PCR product was analyzed by agarose (0.8%) gel electrophoresis, the specific band was extracted from the gel with QIAquick Gel Extraction Kit (QIAGEN, 28706) and analyzed by Sanger sequencing. Furthermore, the presence of the HA-tagged L protein in infected HEK293T (ATCC- CRL-3216) cells was confirmed by western blot analyses with anti-HA antibody.

### Affinity purification mass spectrometry

Ten subconfluent 15-cm dishes of HEK293 FlpIn TREx cells were infected with Cl13_L-HA_ at a MOI of 0.01. Additionally, ten sub-confluent 15 cm dishes of HEK293 FlpIn TREx cells (no bait control) or Strep-HA-GFP expressing HEK293 TREx Flp-In (GFP control) were infected with untagged Cl13 LCMV at a MOI of 0.01. Expression of Strep-HA-tagged GFP in HEK293 TREx Flp-In cell line was induced with doxycycline (1 μg/mL) 24 hours before infection. Cells were harvested 36 hours post infection and lysed for 30 min on ice in the IP buffer (HEPES 50 mM, pH 8.0; NaCl 150 mM, EDTA 5 mM, NP-40 0.5%, NaF 50 mM, Na_3_VO_4_ 1 mM, PMSF 1 mM, protease inhibitors (P8849, Sigma)) and cleared by centrifugation. Affinity purification was performed with anti-HA Clone 7 agarose beads (A2095, Sigma) on Biospin column (732–6008, BioRad) using 50mg of total protein lysate. The flow-through was removed by gravity flow and anti-HA agarose beads were washed 6 times with washing buffer (HEPES 50 mM, pH 8.0; NaCl 150 mM, EDTA 5 mM), the protein complexes were eluted with 100 mM formic acid, neutralized with triethylammonium bicarbonate (TEAB) and digested with trypsin as described previously [47]. The input was normalized based on western blot signal and samples were analyzed by Liquid Chromatography Mass Spectrometry (LC–MS/MS) on a linear trap quadrupole (LTQ) Orbitrap Velos mass spectrometer (ThermoFisher Scientific) coupled to an Agilent 1200 HPLC nanoflow system (Agilent Biotechnologies). Details of the instrument configuration and methodology are described elsewhere [48].

### Co-immunoprecipitation

HEK293T cells were either infected with either Cl13_L-HA_ or untagged Cl13 or transfected either with L-HA encoding plasmid or empty vector control with Effectene Transfection reagent (QIAGEN, 301425) in 6 well plates. 36 hours post infection or transfection cells were harvested, lysed for 30 min on ice in the IP buffer, and then the supernatant was cleared by centrifugation. Co-immunoprecipitation of HA-tagged L protein and PABPC1 and DDX3X was performed using anti-HA agarose beads, Clone 7 (A2095, Sigma). Total cell lysate was incubated with anti-HA agarose overnight at 4°C. Beads were washed 6 times with IP buffer, proteins were eluted with 4% SDS Laemmli sample buffer at 95°C for 10 min and analyzed by western blot.

For reverse co-immunoprecipitation experiments HEK293T cells were transfected with L-HA and/or TRIM21-V5 encoding plasmids with Effectene Transfection reagent (QIAGEN, 301425) in 6 well plates. TRIM21 cDNA was synthesized using the following primers: 5`caccatggcttcagcagc`3 and 5`atagtcagtggatccttgtgatcca`3 and subcloned with pENTR/D-TOPO cloning kit into a plasmid backbone originating from the pTRACER-V5 plasmid (Invitrogen) according to the manufacturer’s protocol (Thermo Fisher Scientific, 450218). L Cl13 LCMV ORF was obtained from the plasmid described previously [45] using the following primers: 5`caccgccatggatgaaatcatctcagaattgagag`3 and 5`gtcgatgtcctcggccacc`3 subcloned by Gateway cloning (Thermo Fisher Scientific) into a plasmid pTO-SII-HA according to the manufacturer’s protocol (Thermo Fisher Scientific, 450218).

36 hours post transfection cells were harvested, lysed for 30 min on ice in the IP buffer, and then the supernatant was cleared by centrifugation. Co-immunoprecipitation of HA-tagged L protein and V5-tagged TRIM21 was performed using anti-V5 agarose affinity gel, Clone V5-10 (A7345, Sigma). Total cell lysate was incubated with anti-V5 agarose affinity gel overnight at 4°C. Beads were washed 2 times with IP buffer, proteins were eluted with 4% SDS Laemmli sample buffer at 95°C for 10 min and analyzed by western blot.

### Western blot

Protein concentration of cell lysates was determined with Pierce Coomassie (Bradford) Protein Assay kit (Thermo Fisher Scientific, 23200). Proteins were analyzed by SDS-Page (Thermo Fisher Scientific, EA0375BOX) using Westran Clear signal PVDF membranes (Sigma Aldrich, 0485289) and the following antibodies: anti-HA.11 epitope tag antibody (Covance, MMS-101P), anti-DDX3X (Bethyl Laboratories, A300-474A), anti-PABPC1 (Cell Signaling, 4992), anti-TRIM21 (New England BioLabs, 92043) and anti-V5 Clone 5C5 (gift from M. Busslinger). The protein size was determined with a Spectra Multicolor High Range Protein Ladder (Thermo Fisher Scientific, 26625). Signal was detected with Pierce ECL Western blotting substrate (Thermo Fisher Scientific, 32209), or Amersham ECL select Western blotting detection reagent (GE Healthcare Life Sciences, RPN2235). Visualization was performed with the chemiluminescent gel documentation system MF-chemi 3.2 Pro (DNR Bio-Imaging Systems) respectively with the Bio-Rad ChemiDoc XRS Gel Documentation system (Bio-Rad Laboratories).

### CRISPR-Cas9 engineering of targeted cells and functional screen

CRISPR-Cas9 single guide RNAs (sgRNAs) for selected target genes were designed using the CRISPR Design Tool [49], sgRNA Designer [50] and E-CRISPR v4.2 [51] online tools based on the target genome sequences obtained from Ensembl genome browser or merged Ensembl/Havana transcripts. Complementary oligo sgRNAs (**S4 Table**) were cloned into *Bsm*BI restriction site of LentiCRISPRv2 [52] (Addgene ID 49535) that was used for lentivirus particle production. To validate the genome editing in the targeted cells, we performed T7EI assays. PCR products were denatured, then temperature was reduced to 25°C. Hybridized PCR products were digested with T7 endonuclease 1 (NEB) for 20 min at 37°C in NEBuffer 2 (NEB, USA) and analyzed by agarose gel electrophoresis (2%). ImageJ 2.0 software was used to quantify the band intensities. PCR products were analyzed by Sanger sequencing and insertion or deletion (Indel) rates were assessed by the TIDE approach [53] using DNA of non-target control cells as a reference control. The non-target control cells were transfected with the empty plasmid, that could not lead to genome editing.

CRISPR-Cas9 engineered HeLa S3 cells (ATCC CLL-2.2) were infected with 0.01 of MOI of LCMV Cl13 WT and supernatant was harvested at 36 hours post infection followed by viral load analyses by FFA. All values were normalized to the non-target control and Log2 transformed.

### Reconstitution of TRIM21-targeted cells

CRISPR-Cas9 TRIM21-targeted HeLa S3 cells were transfected with either TRIM21-encoding plasmid or with mock control (empty vector—EV) with Effectene Transfection reagent (QIAGEN, 301425). 36 hours after transfection cells were infected with either wild type LCMV strain ARM or Cl13 at a MOI of 0.01. Supernatants were harvested 36 hours after infection and viral titers were quantified by focus forming assay. Obtained values were normalized to the non-target control transfected with EV plasmid and Log2 transformed.

### Real-time PCR

Total RNA was extracted using QIAzol lysis reagent (QIAGEN, 79306) and reverse-transcribed with First Strand cDNA synthesis kit (Thermo Fisher Scientific, K1622) according standard protocol. Gene expression by real-time PCR was analyzed using Taqman Fast Universal Master Mix (Thermo Fisher Scientific, 4352042) and Taqman Gene Expression assays for Trim21 (Thermo Fisher Scientific, Hs00989229_g1) and for LCMV NP as described previously [54]. Gene expression data was normalized to the housekeeping gene HPRT1 (Thermo Fisher Scientific, Hs99999909_m1).

### Proteomic analysis

For protein identification the RAW MS data files were converted into Mascot generic format (.mgf) using Proteowizard software v2.1.2708 [55] and searched against the human SwissProt protein database (v. 2013.01) using the two search engines, Mascot (v2.3.02, MatrixScience, London, UK) [56] and Phenyx (v2.6, GeneBio, Geneva, Switzerland) [57]. The search parameters were set with carbamidomethyl cysteine and oxidized methionine as fixed and variable modifications, respectively. One missed tryptic cleavage site was allowed. The Mascot and Phenyx identifications were combined and filtered using the previously described procedure [21] at a <1% protein false discovery rate (FDR).

Known MS contaminants such as trypsin and keratin were discarded from the results and further filtering of proteins specifically binding to LCMV L protein was achieved by comparing L protein pulldowns with the negative controls (GFP and no HA-tagged bait pulldowns) using Top3 quantitation [58] calculated with Skyline v3.1 [59] and SAINTexpress AP-MS filtering software [60]. Based on Top3 quantitation and an average of SAINT probabilities (AvgP) >0.95, all potential interactors with >120 fold increase in abundance in L pulldowns were considered to be high-confidence interactors. The visualization of the obtained networks was performed in Cytoscape v.3.4.0 [61].

### Gene ontology and pathway enrichment analysis

The obtained datasets for the L protein LCMV interactome were integrated with publicly available data on host-virus interactions for other viral RdRps [22] (**S3 Table**). Functional gene annotations were obtained from the Gene Ontology (GO) database [62]. Gene set enrichment analyses were performed using Fisher’s exact test with all approved protein coding genes from the HUGO Gene Nomenclature Committee (HGNC) [63] as background set. The resulting *p*-values were corrected for multiple hypotheses testing according to the Bonferroni procedure using a cut-off of p-value<0.05. For the visualization of the resulting GO terms we used ReviGO [64].

### Functional protein modules clustering

In order to identify groups of proteins with similar function we used the following network-based approach: First, we quantified the functional similarity of all protein pairs from the overlap of their respective GO annotations using the Tanimoto coefficient: $T=\frac{c}{a+b-c}$, where “c” gives the number of shared annotations and “a” and “b” the number of annotations of the two respective proteins. Next, we connected all protein pairs whose functional similarity exceeded a threshold of *T* ≥ 0.1, resulting in a network of 797 human proteins, 5 viral RdRps and 1033 links. We finally used a community detection algorithm from network theory [65] to identify groups of highly interconnected proteins, i.e. proteins with a high degree of functional coherence. Each community was manually assigned a functional module name based on the most prevalent GO-terms (**S5 Table**). The visualization of the network was performed in Cytoscape v.3.4.0 [61].

### Network analysis

The human host proteome we used was manually curated in [23] and contains 13,460 proteins connected by 141,296 physical interactions. To quantify the extent to which a set of viral targets is localized within a certain neighborhood of the host proteome, we analyzed their largest connected component (LCC), i.e., the highest number of viral targets that are directly connected to one another [23]. To test the statistical significance of a measured LCC size, we compared it to the control distribution obtained from 100,000 random simulations in which gene sets of the same size were chosen completely at random from the network.

### Statistical analyses and data visualization

Two-tailed Student’s t-tests were used for comparisons between two groups, one-way ANOVA with Bonferroni correction were used for comparisons between multiple groups. The values shown in the line and bar graphs indicate the mean +/- standard error of the mean. Significant p-values are indicated as follows: * p≤0.05, ** p≤0.01, *** p≤0.001, **** p≤0.0001. Statistical analyses and graph preparation were performed in GraphPad Prism (version 6.0b).

### Ethics statement

Experiments conducted at the animal facility of the Medical University of Vienna were approved by the ethical committee of the Medical University of Vienna and the Austrian Federal Ministry of Science and Research with the animal protocol number 66.009/0318-II/3b/2012 in accordance with the Austrian law for animal experiments (TVG, BGBl. Nr. 501/1989 i.d.F. BGBI. I Nr. 162/2005). Mouse experiments performed at the University Basel were approved by the Veterinary Office of the Canton on Basel (#2665/28404) and were performed in accordance with the Swiss law for animal protection (TSchG 455).

## Supporting information

S1 Fig Functional validation of Cl13_L-HA_ virus and HA-immunoprecipitation. **(A**) HEK293T cells were infected at a MOI of 3 with either Cl13_L-HA_ or with untagged Cl13. Cells were harvested and lysed at 36 hours post infection for western blot analyses with anti-HA antibodies. (**B**) C57BL/6J mice were infected with 2x10^6^ FFU either Cl13_L-HA_ or untagged Cl13 and spleen samples were analyzed 50 days post infection by Sanger sequencing. (**C**) Fractions from one-step purification of Cl13_L-HA_ protein were collected during the mass spectrometry sample preparation and analyzed by western blot with antibodies specific to HA and NP LCMV. NP, as a known L interactor, was used as a positive control to confirm the successful immunoprecipitation of L-HA. Percentage indicates the amount of each fraction collected during AP-MS pulldown preparation loaded on the gel. (**D**) HEK293T cells were infected with MOI 3 either with Cl13_L-HA_ containing either L1079K or L1079Q, or untagged virus. Cells were harvested and lysed 36 hours post infection and co-immunoprecipitation was performed with anti-HA followed by western blot analyses with antibodies specific to the endogenous proteins DDX3X, PABPC1 and TRIM21 as well as HA. IP–immunoprecipitation. **(E)** HEK293T cells were transfected with plasmid encoding HA-tagged L protein or empty vector control. Cells were harvested and lysed 36 hours post transfection and co-immunoprecipitation was performed with anti-HA followed by western blot analyses with antibodies specific to the endogenous proteins DDX3X, PABPC1 and TRIM21. **(F)** HEK293T cells were transfected with plasmid encoding HA-tagged L protein and/or V5-tagged TRIM21. Cells were harvested and lysed 36 hours post transfection and co-immunoprecipitation was performed with anti-V5 followed by western blot analyses with antibodies specific to the HA and V5. * marks a non-specific protein band.
(TIF)

S2 Fig Confirmation of the genome editing for CRISPR-Cas9 targeted cells. Confirmation of the genome editing for CRISPR-Cas9 targeted cells using (**A**) T7EI cleavage assay followed by the band intensity quantification with ImageJ software and (**B**) Sanger sequencing followed by tracking of indels by decomposition (TIDE) quantification. For TIDE analyses primers were designed covering the respected targeted region using Ensembl genome browser or merged Ensembl/Havana transcripts to PCR-amplify the selected region. To evaluate indel frequencies we used non-target control treated sample (transfected with an empty plasmid) as a reference control. Bars represent indel frequencies for each cell line. (**C**) TRIM21 and DDX3X CRISPR-Cas9 targeted cells were lysed and analyzed by western blot with antibodies specific to the endogenous TRIM21 or DDX3X and actin.
(TIF)

S3 Fig *TRIM21* expression in HEK293T cells infected with Cl13 LCMV. HEK293T cells were infected with LCMV Cl13 WT at a MOI of 3 and harvested at the indicated time points. The gene expression for NP of LCMV and *TRIM21* was measured by RT-PCR. The arbitrary units were calculated using HPRT1 as a housekeeping gene, then fold change for each gene was calculated using 0 hpi as a reference point for *TRIM21* and 2 hpi for NP LCMV.
(TIF)

S4 Fig Analyses of the L protein of LCMV strain ARM *in vitro* and *in vivo*. **(A)** Two independently generated HeLa S3 CRISPR-Cas9 targeted cell pools per gene of interest for 5 genes were infected in triplicate wells with LCMV ARM WT at a MOI of 0.01 and viral loads were measured at 36 hours post infection by focus forming assay. The obtained data were normalized to the non-target control and log2 transformed. (**B**-**C**) C57BL/6 and *Trim21*^-/-^ mice were infected with 2x10^6^ FFU of LCMV ARM WT. Viral titers were determined in (**B**) blood at indicated time points and in (**C**) organs at 21 days post infection. Each symbol and bar represents the mean ± SEM of three to five mice. Statistical significance was calculated by unpaired t-test (**B**) or by two-way ANOVA (**C**). Significant p values were indicated as follows: ns—non significant, * p≤0.05, ** p≤0.01, *** p≤0.001, **** p≤0.0001.
(TIF)

S1 Table Primers used for reverse genetic engineering of L protein-tagged LCMV. (XLSX)

S2 Table Mass spectrometry data. (XLSX)

S3 Table Host protein interactomes of viral RdRps from public databases. (XLSX)

S4 Table sgRNA oligonucleotide sequences. (XLSX)

S5 Table Protein functional modules targeted by L protein and other RdRps. (XLSX)
