# Supplementary figures and images for: Characterization of host proteins interacting with the lymphocytic choriomeningitis virus L protein
Source: PLoS Pathog. 2017 Dec 20;13(12):e1006758. doi: 10.1371/journal.ppat.1006758 (PMC5738113; doi:10.1371/journal.ppat.1006758)

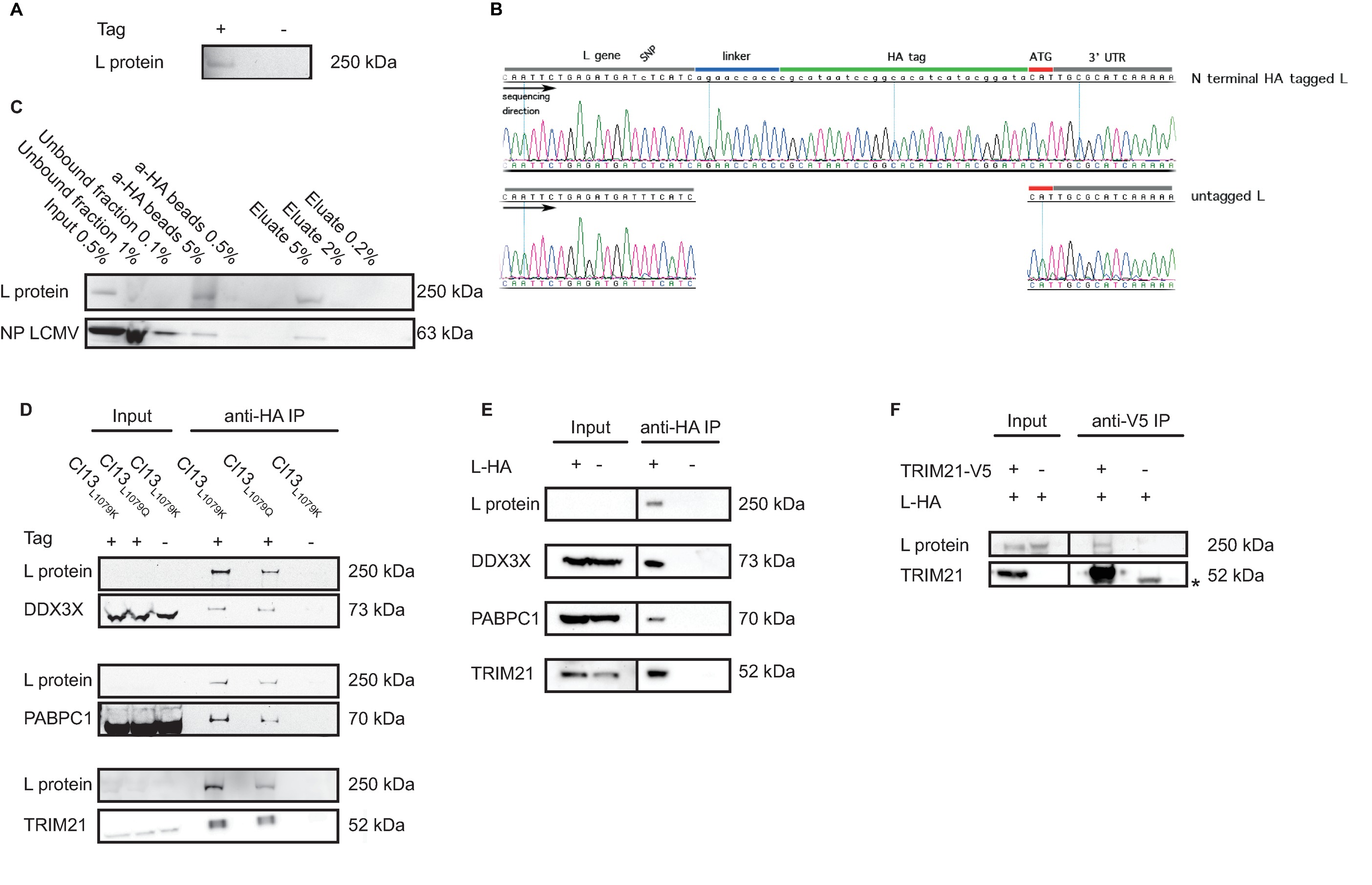

Supplement: S1 Fig — (A) HEK293T cells were infected at a MOI of 3 with either Cl13L-HA or with untagged Cl13. Cells were harvested and lysed at 36 hours post infection for western blot analyses with anti-HA antibodies. (B) C57BL/6J mice were infected with 2x10^6^ FFU either Cl13L-HA or untagged Cl13 and spleen samples were analyzed 50 days post infection by Sanger sequencing. (C) Fractions from one-step purification of Cl13L-HA protein were collected during the mass spectrometry sample preparation and analyzed by western blot with antibodies specific to HA and NP LCMV. NP, as a known L interactor, was used as a positive control to confirm the successful immunoprecipitation of L-HA. Percentage indicates the amount of each fraction collected during AP-MS pulldown preparation loaded on the gel. (D) HEK293T cells were infected with MOI 3 either with Cl13L-HA containing either L1079K or L1079Q, or untagged virus. Cells were harvested and lysed 36 hours post infection and co-immunoprecipitation was performed with anti-HA followed by western blot analyses with antibodies specific to the endogenous proteins DDX3X, PABPC1 and TRIM21 as well as HA. IP–immunoprecipitation. (E) HEK293T cells were transfected with plasmid encoding HA-tagged L protein or empty vector control. Cells were harvested and lysed 36 hours post transfection and co-immunoprecipitation was performed with anti-HA followed by western blot analyses with antibodies specific to the endogenous proteins DDX3X, PABPC1 and TRIM21. (F) HEK293T cells were transfected with plasmid encoding HA-tagged L protein and/or V5-tagged TRIM21. Cells were harvested and lysed 36 hours post transfection and co-immunoprecipitation was performed with anti-V5 followed by western blot analyses with antibodies specific to the HA and V5. * marks a non-specific protein band. (TIF) [file ppat.1006758.s001.tif]

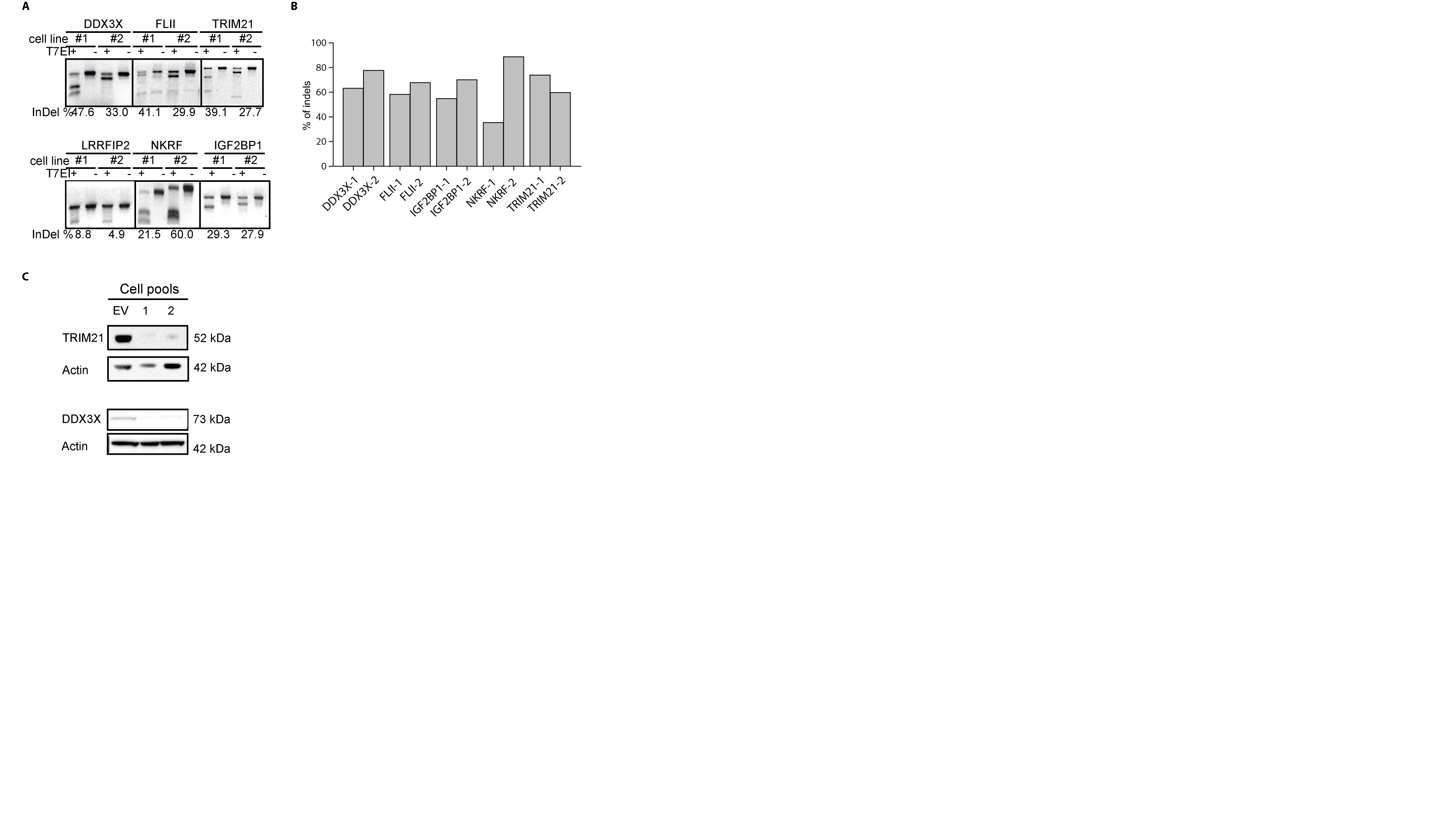

Supplement: S2 Fig — Confirmation of the genome editing for CRISPR-Cas9 targeted cells using (A) T7EI cleavage assay followed by the band intensity quantification with ImageJ software and (B) Sanger sequencing followed by tracking of indels by decomposition (TIDE) quantification. For TIDE analyses primers were designed covering the respected targeted region using Ensembl genome browser or merged Ensembl/Havana transcripts to PCR-amplify the selected region. To evaluate indel frequencies we used non-target control treated sample (transfected with an empty plasmid) as a reference control. Bars represent indel frequencies for each cell line. (C) TRIM21 and DDX3X CRISPR-Cas9 targeted cells were lysed and analyzed by western blot with antibodies specific to the endogenous TRIM21 or DDX3X and actin. (TIF) [file ppat.1006758.s002.tif]

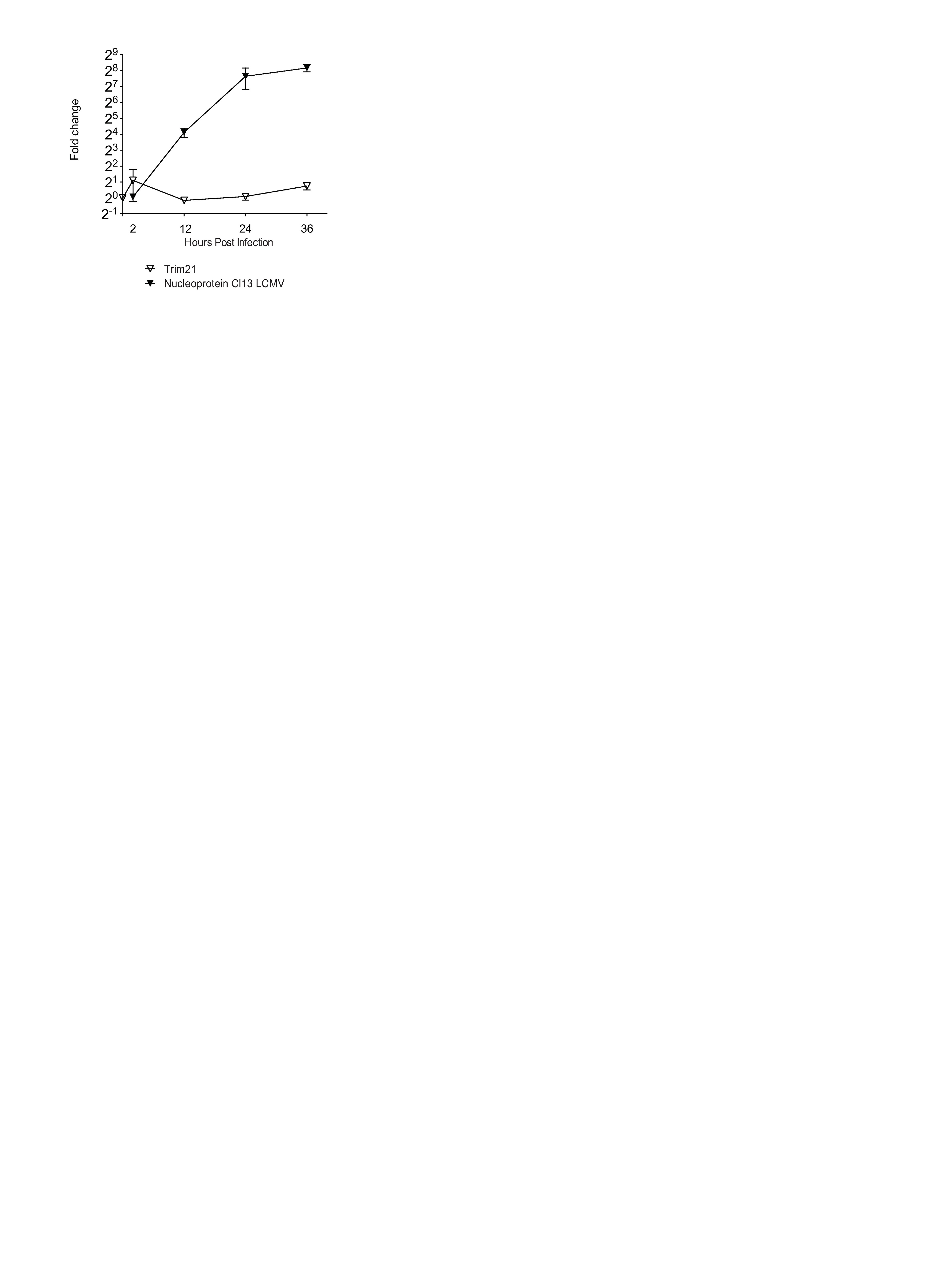

Supplement: S3 Fig — HEK293T cells were infected with LCMV Cl13 WT at a MOI of 3 and harvested at the indicated time points. The gene expression for NP of LCMV and TRIM21 was measured by RT-PCR. The arbitrary units were calculated using HPRT1 as a housekeeping gene, then fold change for each gene was calculated using 0 hpi as a reference point for TRIM21 and 2 hpi for NP LCMV. (TIF) [file ppat.1006758.s003.tif]

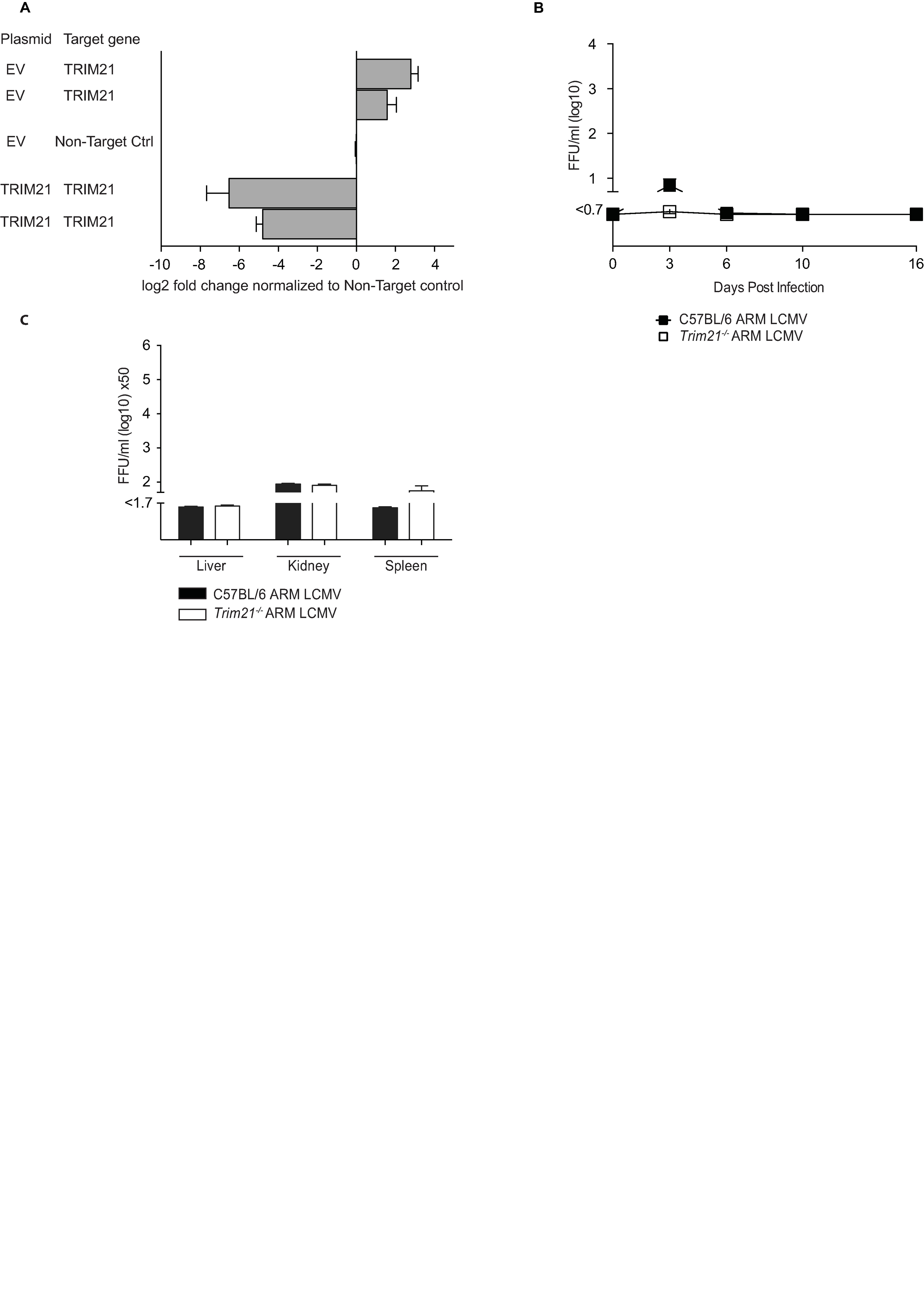

Supplement: S4 Fig — (A) Two independently generated HeLa S3 CRISPR-Cas9 targeted cell pools per gene of interest for 5 genes were infected in triplicate wells with LCMV ARM WT at a MOI of 0.01 and viral loads were measured at 36 hours post infection by focus forming assay. The obtained data were normalized to the non-target control and log2 transformed. (B-C) C57BL/6 and Trim21-/- mice were infected with 2x10^6^ FFU of LCMV ARM WT. Viral titers were determined in (B) blood at indicated time points and in (C) organs at 21 days post infection. Each symbol and bar represents the mean ± SEM of three to five mice. Statistical significance was calculated by unpaired t-test (B) or by two-way ANOVA (C). Significant p values were indicated as follows: ns—non significant, * p≤0.05, ** p≤0.01, *** p≤0.001, **** p≤0.0001. (TIF) [file ppat.1006758.s004.tif]
